# Human adenovirus binding to host cell receptors: a structural view

**DOI:** 10.1007/s00430-019-00645-2

**Published:** 2019-11-29

**Authors:** Aleksandra Cecylia Stasiak, Thilo Stehle

**Affiliations:** 1 Interfaculty Institute of Biochemistry, University of Tübingen, Hoppe-Seyler-Str. 4, 72076 Tübingen, Germany; 2 Department of Pediatrics, Vanderbilt University School of Medicine, Nashville, TN 37232 USA

**Keywords:** Human adenovirus, Structural biology, X-ray crystallography, Cryoelectron microscopy, Review

## Abstract

Human Adenoviruses (HAdVs) are a family of clinically and therapeutically relevant viruses. A precise understanding of their host cell attachment and entry mechanisms can be applied in inhibitor design and the construction of targeted gene delivery vectors. In this article, structural data on adenovirus attachment and entry are reviewed. HAdVs engage two types of receptors: first, an attachment receptor that is bound by the fibre knob protein protruding from the icosahedral capsid, and next, an integrin entry receptor bound by the pentameric penton base at the capsid vertices. Adenoviruses use remarkably diverse attachment receptors, five of which have been studied structurally in the context of HAdV binding: Coxsackie and Adenovirus Receptor, CD46, the glycans GD1a and polysialic acid, and desmoglein-2. Together with the integrin entry receptors, they display both symmetrical and asymmetrical modes of binding to the virus as demonstrated by the structural analyses reviewed here. The diversity of HAdV receptors contributes to the broad tropism of these viruses, and structural studies are thus an important source of information on HAdV-host cell interactions. The imbalance in structural data between the more and less extensively studied receptors remains to be addressed by future research.

## Introduction

### Human adenoviruses as pathogens and therapeutics

Human Adenoviruses (HAdVs) are a family of non-enveloped double-stranded deoxyribonucleic acid (dsDNA) viruses with genomes of about 35 kilobases (kb) [1]. They are causative agents of a wide range of illnesses, such as conjunctivitis, gastroenteritis and respiratory infections [2]. Over 100 types of HAdVs, classified into seven groups (A–G), have been reported to the HAdV Working Group (http://hadvwg.gmu.edu/). Viruses among these groups vary in pathology and molecular characteristics, for instance receptor specificity and host cell tropism [3, 4].

Due to their large genome capacity and the ability to infect different cell types, HAdVs have been extensively studied as gene delivery vectors, and HAdV family members have been used in hundreds of clinical trials in oncology, gene therapy, and vaccinology [5, 6]. A detailed understanding of HAdV host cell attachment and entry mechanisms facilitates the design of vectors with a particular tropism, as well as the rational design of antiviral compounds. Structural biology techniques, in particular, can provide information on the molecular details of virus binding to host cell, and can identify strategies for the disruption of such interactions. So far, structural analyses of HAdV particles and their components have revealed symmetrical and asymmetrical binding modes to HAdV receptors, and these will be reviewed below.

### HAdV attachment and entry

**Fig. 1:**
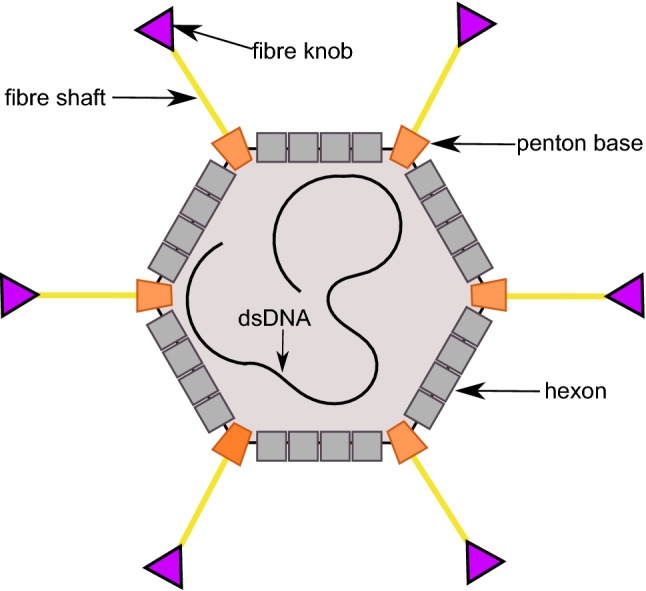
A schematic drawing of Human Adenovirus, showing the major capsid proteins that interact with receptors. The fibre knob is in purple, the fibre shaft in yellow, the penton base in orange and the hexon in grey. The minor capsid proteins and non-structural proteins have been omitted for simplicity

The HAdV capsid possesses *T* = 25 icosahedral symmetry, and consists of three major proteins: the hexon, the penton base and the fibre (see Fig. 1) [2, 7]. Both the fibre and penton base, forming the penton complex at the vertices of the icosahedron, can engage host cell receptors. The fibre can be divided into a C-terminal, globular structure, the “knob”, which protrudes away from the capsid and mediates the initial interaction with attachment receptors, and an N-terminal, elongated “shaft” that anchors the fibre into the viral capsid. Binding of the knob to attachment receptors is followed by the penton base binding to the integrin entry receptor.

The fibre is a homotrimeric structure of between 60–80 kDa per monomer and its globular knob can engage different receptors [8–12]. The monomeric C-terminal fibre knob contains an eight-stranded antiparallel β-sandwich fold, comprised of two β-sheets, with multiple loops. The trimeric propeller-like knob is formed by intertwining β-sheets and has a deep depression at the centre, which thins into narrowing channels, exposing around 65% residues to the solvent [8, 13]. The knob is mounted on the shaft, which consists of repeats of a hydrophobic 15-residue sequence assembled into a trimeric β-spiral [14]. The number of repeats, and hence the length of the shaft, varies between HAdV types. Residues at the very N-terminal region of the fibre knob form a tail that interacts non-covalently with the penton base [15].

Multiple HAdV attachment receptors have been identified: the cell surface proteins Coxsackie and Adenovirus Receptor (CAR) [16, 17], CD46 [10], and desmoglein-2 (DSG-2) [9], as well as the glycans GD1a [11] and polysialic acid [18]. The interactions of all five receptors with HAdV have been established using structural biology techniques and are the focus of this review. Other adenovirus receptors, such as heparan sulphate glycosaminoglycans [4], or factors IX [19] and X [20], have also been described, but we currently lack detailed structural information about their modes of binding to the virus.

Once HAdV has attached to the cell surface, the fibre starts disassociating from the capsid, exposing the penton base [21]. The penton base forms the vertex pentamer (see Fig. 1), which binds to the integrin entry receptor and exploits integrin-mediated signalling to enter the cell by endocytosis. HAdVs have been shown to use multiple types of integrins as their receptors, again highlighting their broad tropism [22].

The penton monomer consists of two domains: a jelly-roll domain proximal to the virion centre, and a distal insertion domain [23]. The latter contains the variable, highly mobile RGD loop, so named because it contains an arginine-glycine-aspartic acid tripeptide sequence motif. The integrin-binding RGD motif mediates binding between the integrin and the penton base, with the exception of group F HAdV-40 and -41, where the interaction is presumed to take place in another manner due to the lack of this motif [24]. The length of the RGD loop varies significantly between strains, ranging from 36 amino acids for HAdV-12 to 99 amino acids for HAdV-5.

## HAdV Interactions with attachment receptors

### Coxsackie and adenovirus receptor (CAR)

CAR and CD46 have been the most extensively studied HAdV receptors, and their interactions with the fibre knob are particularly well understood. The first structure of a fibre knob bound to CAR was published in 1999, showing that the N-terminal domain of CAR engages the fibre knob with an unusually discontinuous interface that features large, solvent-filled areas [8]. Structural information about fibre knob binding to CD46 became available in 2007, revealing a binding interface that spans the N-terminal two CD46 domains and that likely alters the conformation of unliganded CD46 [25]. The interactions of the fibre knobs with CAR and CD46 have been reviewed, for example, in [26]. Given the lack of extensive developments since then, this review will only give a brief summary.

CAR is a transmembrane protein mediating cell–cell adhesion [27]. It is a member of the Junctional Adhesion Molecule (JAM) family, and present in tight junctions and on epithelial cells at their lateral surfaces. CAR can form homodimers [28] or heterodimers with numerous extracellular and intracellular proteins, for instance fibronectin and JAM-L [29]. These interactions have been linked to processes such as T cell activation and cell adhesion, among others. CAR is a high-affinity receptor for HAdV groups A and C–F, as well as Coxsackievirus group B viruses [27].

**Fig. 2:**
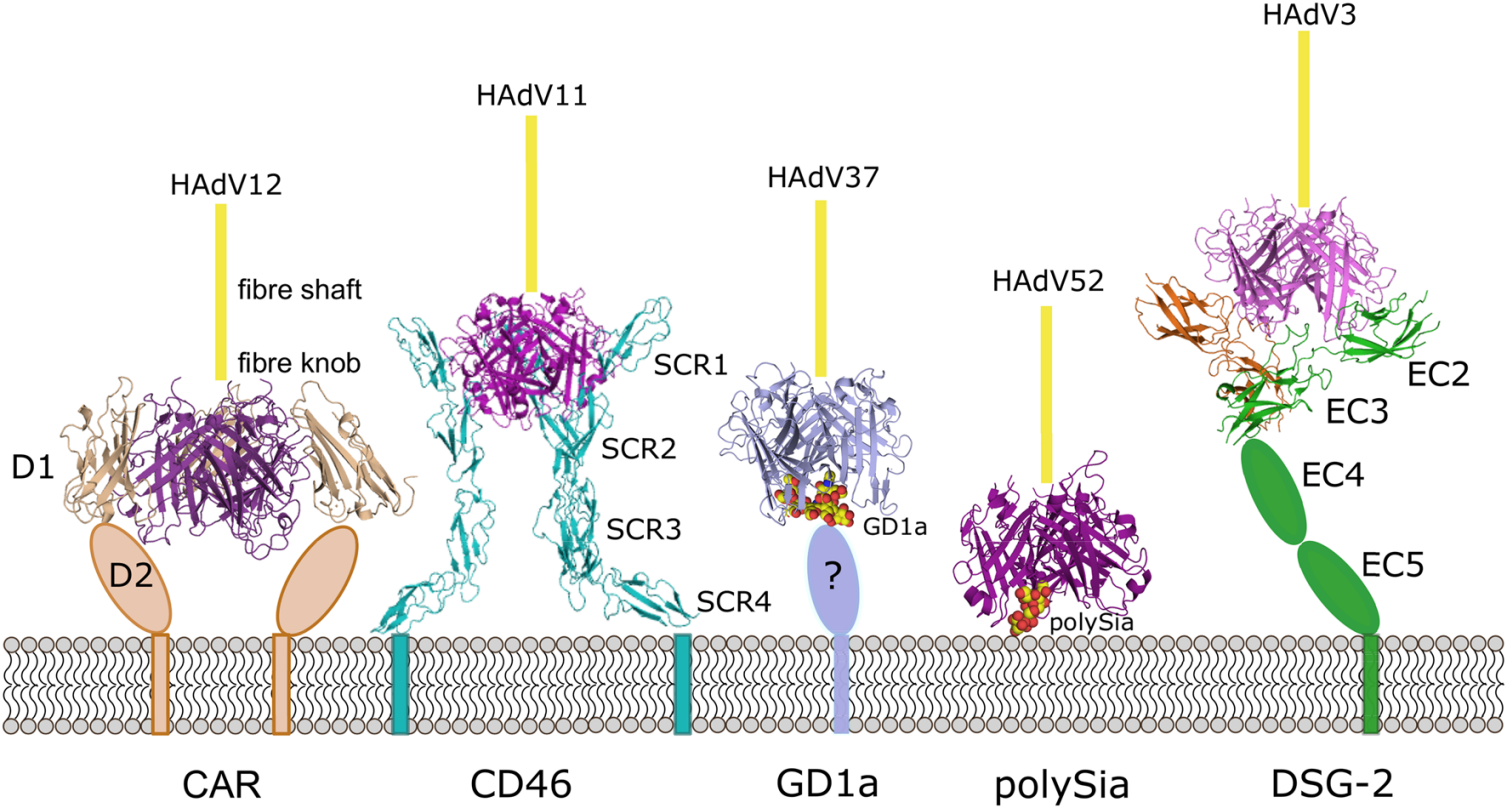
Human adenovirus knob binding to attachment receptors. The trimeric knob is shown in shades of purple, the trimeric fibre shaft is represented by a yellow line (length not to scale). Domains not featured in structures are represented with cartoon shapes. CAR-D1 is bound to HAdV-12, PDB ID: 1KAC [8], CD46 to HAdV-11, PDB ID: 3O8E [39], GD1a to HAdV-37, PDB ID: 3N0I, [11], polySia to HAdV-52, PDB ID: 6G47 and two copies of DSG-2 to HAdV-3, PDB ID: 6QNU

CAR comprises two immunoglobulin-like extracellular domains, CAR-D1 and CAR-D2, both of which have been characterised structurally [28, 30]. The D1 domain forms direct contacts with the fibre knob. The crystal structure of D1 in complex with the knob from HAdV-12 shows one D1 domain bound per knob monomer, with binding occurring at the interface of two monomers rather than at the central cavity (see Fig. 2) [8]. Interactions are mediated by four flexible loops of the knob, as was shown by both structural and mutational studies [31–33]. The AB loop of the fibre knob is responsible for over a half of the protein–protein contacts and is, therefore, an important determinant of CAR receptor specificity [8].

In a recent publication, the structures of the fibre knobs of HAdV-26 and 48 (group D) were solved, and computer modelling, surface plasmon resonance (SPR) and inhibition assays were used to examine binding to potential receptors, primarily CAR, CD46 and DSG-2 [34]. The structure of the fibre knob bound to CAR and CD46 was simulated in silico using homology models. A limited flexibility of loops involved in receptor binding in CAR and CD46 was postulated based on the X-ray data and interaction calculations. Binding of a lower affinity to CAR and no significant binding to CD46 and DSG-2 were proposed based on loop conformation and this was supported by competition inhibition assays.

### CD46 (human membrane cofactor protein)

Human CD46, or membrane cofactor protein (MCP), is a ubiquitously expressed transmembrane glycoprotein involved in multiple processes, such as complement response inhibition, fertilisation, and adaptive immunity regulation through its extracellular domains [35, 36]. CD46 is a member of the RCA (regulators of complement activation) family, and as such its role in binding complement proteins C3b and C4b is of particular importance since this interaction prevents the activation of complement response against autologous cells [36]. Complement system proteins are also pathogen receptors. CD46 can serve as a receptor for pathogenic bacteria and viruses, including *Neisseria*, measles, human herpesvirus-6, and some group B HAdVs [36]. RCA family members CD55 and CD21 serve as receptors for echoviruses [37] and Epstein-Barr virus [38], respectively.

The extracellular part of CD46 consists of four short consensus repeats (SCR, 1–4) and a serine, threonine and proline (STP)-rich region [39]. In an unbound form, SCR4 (proximal to cell membrane) is bent in relation to the almost-linearly arranged remaining subunits, forming a “hockey stick” shape. SCR1 and 2 have been identified as the domains interacting with the HAdV knob. When only these two subunits are expressed, a greater degree of flexibility is observed between the two domains. However, when interacting with the HAdV-11 knob, SCR1 becomes almost colinear with SCR2 [25]. The interface of CD46 and this knob is remarkably long and flat, with the binding mediated not only by loops but also main-chain interactions and *π*–*π* stacking of crucial arginine residues, including a conserved Arg280 in the knob. HAdV-11 binds to CD46 with high affinity and comparisons between the structure of its fibre knob and that of the lower affinity group B HAdVs (HAdV 7, 14, 21) have provided insight into mechanisms and determinants of the interaction with CD46. These include absence of an Arg residue next to Arg280 involved in the stacking [40] and a change in position of surface loops which disrupt the receptor-virus interface [41]. In all fibre knob-CD46 complexes, the binding is symmetrical and involves one CD46 per one knob monomer. All CD46 molecules make identical contacts with the three fiber knob monomers (see Fig. 2).

### Glycans: GD1a and polysialic acid

Glycans, or carbohydrates, constitute an essential class of molecules that is known to serve a wide range of physiological functions [42]. Glycans can be conjugated to other, non-saccharide moieties, such as proteins or lipids, and their diversity in structure, as well as chemical modifications, enables them to mediate functions in signalling, adhesion, and developmental processes. Their prominent display on many cell surfaces makes them a target for numerous pathogens [42].

The first structural data showing interactions of HAdVs with glycans identified three sialic-acid binding sites at the top of the fibre knobs of both HAdV-37 and HAdV-19p (prototypical), around the central cavity [43]. Sialic acid is a monosaccharide derived from neuraminic acid, which serves a range of functions, for instance in immune regulation, and is commonly found on cell surfaces [44]. Sialic acid-containing molecules are employed as receptors by numerous viruses, including influenza viruses, rotaviruses, polyomaviruses, paramyxoviruses, and HAdVs, primarily from group D [45]. In HAdVs, the sialic acid residues bound were moieties of a sialyl-lactose ligand, and both α(2,3)- and α(2,6)-linked ones could bind the knob. Unlike for CD46 and CAR, the binding sites for sialic acid were located on top of the fibre knob, one per knob monomer [43]. However, identification of the full glycan ligand of HAdV-37 as the GD1a glycan showed that two binding sites could be occupied by a single receptor [11] (see Fig. 2).

GD1a is a ganglioside (a glycosphingolipid) prominent in the mammalian nervous system [46]. The GD1a ganglioside is not the HAdV receptor itself, but rather the GD1a glycan is thought to be attached to a glycoprotein with an up to now unidentified protein conjugate. GD1a is Y-shaped and contains two terminal sialic acids, which engage the fibre knob at the top in a very similar manner for both. The sialic acid carboxylate group forms hydrogen bonds with residue Lys345, which was experimentally shown to be crucial for sialic-acid binding [11]. Residues from two fibre knob monomers are involved in binding one sialic acid moiety: one monomer provides hydrogen bonds and the other contributes van der Waals interactions and water-mediated hydrogen bonds, as well as water-mediated hydrogen bonds to galactose moieties anterior to the sialic acids. These glycan moieties bond to sialic acid pointed upwards, away from the binding site, thus leaving space for longer chains terminating in a sialic acid [11]. Knobs that bind sialic-acid are characterised by a highly positive electrostatic potential, which is thought to complement the negative charges of the receptor. No conformational change is seen on binding [43].

The divalent GD1a receptor engages two out of three possible binding sites in the knob. This asymmetry of binding differentiates the GD1a receptor from CAR and CD46, although it is not a unique feature among the HAdV receptors (see below).

Another HAdV type which uses a glycan as its receptor is HAdV-52, a member of group G, which has two different types of fibres: a short fibre and a long fibre, that are present in a 1:1 ratio on the virion [18]. The knob of the long fibre binds to CAR, while the knob of the short fibre binds polysialic acid, and this latter compound is the major attachment receptor for this virus type. The first crystal structure of short fibre knob in complex with a glycan bound 2-*O*-methyl-sialic acid and showed that the binding site is distinct from the one seen in HAdV-37. It is located where the EG and GH loops from two monomers come into contact, and there are again three identical binding sites per knob. The interactions between the knob and the receptor are hydrogen bond-mediated both by the backbone and side chains of the short fibre knob. The conserved RGN motif on the GH loop is of importance for this, although it seems exclusive to HAdV-52 [47]. The receptor was identified as α(2,8)-linked polysialic acid, and the structure obtained by X-ray crystallography showed the importance of transient electrostatic interactions with long glycan chains for the binding [18].

Polysialic acid (polySia) has been linked to many developmental functions, particularly in the human nervous system [48]. While only the non-reducing end was shown to interact with the knob in a stable manner, less stable and less directed electrostatic interactions were shown to also be of consequence to its binding [18]. A positively-charged border around the binding site and the binding site itself mediates contact with polyanionic sialic acid residues further away from the non-reducing end. Due to the transient nature of these interactions, they were not clearly visible in the structure, but simulations suggested that beyond a fifth sialic acid residue these interactions no longer increase. The ability to bind sialic acid chains of different lengths may also assist in infecting different cell types and hosts. An arginine residue was also shown to be crucial to polySia binding by maintaining the appropriate charge, and three polySia binding was symmetrical.

In addition to the two binding sites described above, it is also worth noting that a third sialic acid binding site has been identified in canine adenovirus 2 [49], again highlighting the diversity and adaptability of the adenovirus fibre knob to variable receptors.

### Desmoglein-2

DSG-2 is a transmembrane glycoprotein that belongs to the cadherin family and is involved in maintaining cell–cell adhesion in structures termed desmosomes, which are particularly important in tissues undergoing significant mechanical stress (e.g. heart muscle) [50]. In these junctions, adhesion is mediated by DSG-2 forming heterodimers with a related protein, desmocollin [51]. DSG-2 overexpression has been observed in a number of cancers, and the protein has been identified as a receptor for some group B HAdVs, that have been classified as group B-2 and include HAdV 3, 7, 11 and 14 [9].

The structure of the extracellular fragment of DSG-2 shows that the protein comprises five extracellular cadherin domains (EC1-5) in addition to a transmembrane segment and an intracellular domain [51]. These five domains (with EC1 distal to the cell membrane, and EC5 the most proximal) are linked linearly by Ca2+ ion-binding regions, and they form a curved shape due to a 100° bend between EC3 and EC4. One side of the protein is extensively glycosylated. In the crystal structure, DSG-2 forms homodimers (unlike the desmocollin-containing heterodimers favoured in vivo), linked by strand-swapping mechanisms [51].

A recently published cryo-electron microscopy (cryoEM) structure of the fibre knob-binding domains (EC2 and EC3) of DSG-2 in complex with the HAdV-3 fibre knob reveals that this interaction is also an example of a non-symmetrical receptor-fibre knob interaction [52]. In fact, two distinct stoichiometries of binding are observed: a 1:1, and a 2:1 receptor to fibre knob ratio (see Fig. 2). Importantly, no 3:1 binding is seen, indicating that the trimeric knob cannot engage three DSG-2 molecules at the same time, perhaps because of steric hindrance.

DSG-2 binds at the centre-top of the fibre knob, and in the 1:1 complex it interacts with two out of three monomers. The interactions are primarily mediated by knob loops, with one DSG-2 monomer contacting one knob monomer. The EC2 domain has a stabilising function, while EC3 participates in most of the interactions. Like for CD46, a positional shift is observed in the DSG-2 fragment on fibre knob binding: EC2 rotates by about 10°, once the first, essential interaction with EC3 has been established.

In the 2:1 complex the binding of the second DSG-2 molecule is mediated by very similar contacts, although the limited resolution (3.5 and 3.8 Å) makes it challenging to identify them and their nature clearly. An Asp261 fibre knob residue which is presumed to stabilise loop conformation was shown to be essential to receptor binding. However, it is doubtful that such 2:1 binding mode would be possible in vivo, among the desmocollin-DSG-2 heterodimers in cell–cell junctions [52].

The structural data on DSG-2 binding to HAdV show that the knob is likely too small, and the binding sites for DSG-2 are too close to each other, to allow for simultaneous binding of three DSG-2 molecules to the same knob. The observed asymmetrical, stoichiometrically non-uniform binding of DSG-2 to the fibre knob is of particular interest to studies of integrin-penton base binding (see below), which is also prone to forming asymmetric complexes of different stoichiometries. This decreases the chances of successfully obtaining crystals for X-ray studies, and increases the challenges of cryo-EM data processing as different forms of the complex need to be processed separately.

## HAdV interactions with entry receptors—proteins of the integrin family

**Fig. 3:**
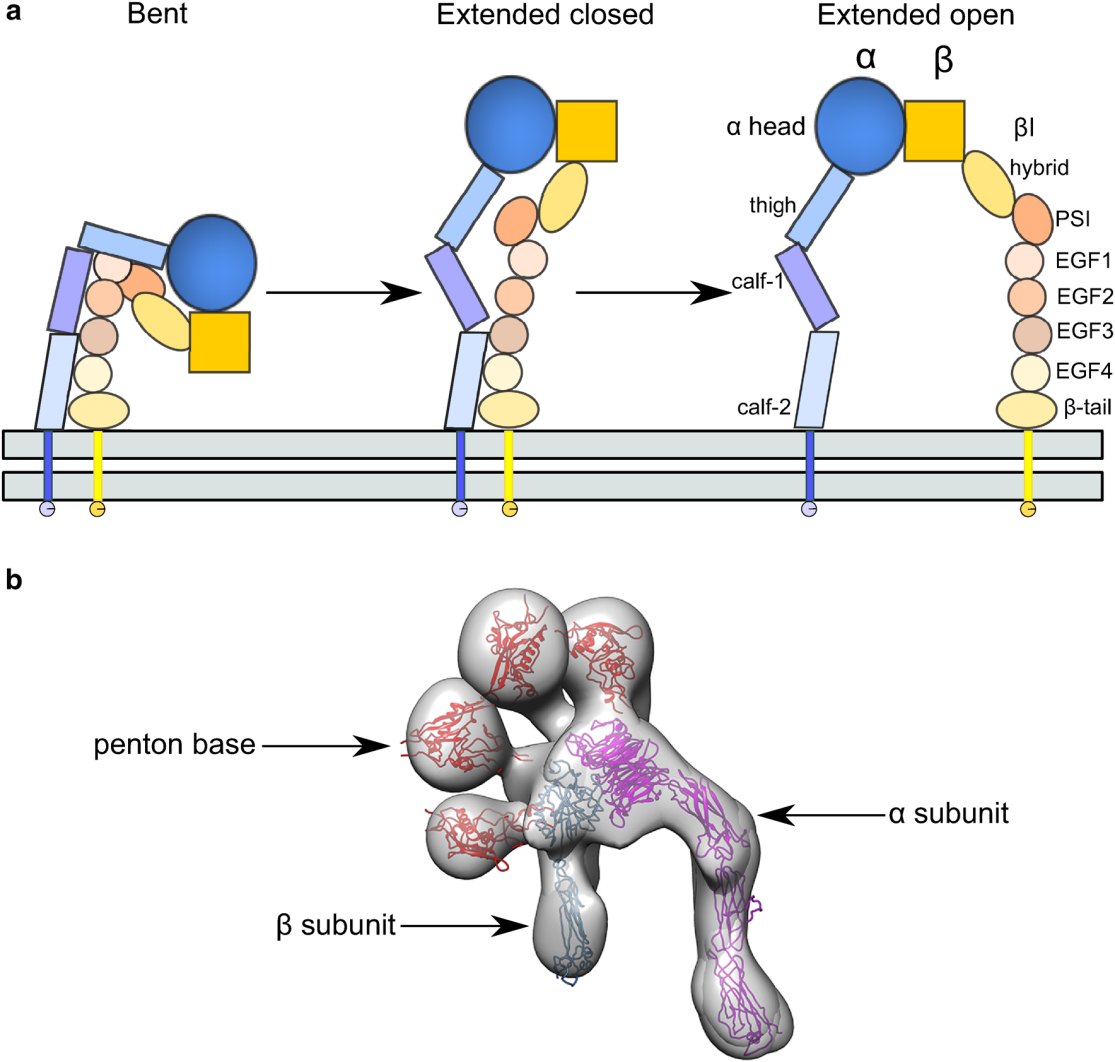
**a** A cartoon representation of integrin conformational changes. **b** The different binding modes for HAdV-9 monomeric penton base to integrin α_v_β_3_. The penton base is in red, the integrin α subunit is in purple, and the β subunit in blue. EMDB IDs: 5955–5973 [58]

Integrins are a family of heterodimeric transmembrane proteins involved in signalling between the cell and its environment. They influence processes such as growth, development, immunity, and have been implicated in cancer [53]. Integrins consist of two extracellular subunits, the α and β chains, which each have single transmembrane domains and short cytoplasmic tails [54]. Eighteen different α and eight different β subunits have been identified in vertebrates, combining into 24 proteins. These subunits are composed of a number of smaller domains, which enable a remarkable degree of conformational flexibility of the protein: from a bent to an extended open conformation (see Fig. 3a). This shift is associated with activation of signalling [53].

Due to their ubiquity, integrins are exploited by a number of viruses as receptors, including the foot-and-mouth disease virus, members of the herpes virus family, reoviruses and the human papillomavirus-16 [55]. Adenoviruses also employ integrins as entry receptors. They engage integrins via the penton protein, and a penton-integrin interaction following attachment seems to be uniform among HAdVs. Interactions with integrins stimulate integrin clustering signalling for endocytosis, and virus entry into the host cell via the endocytic pathway is thought to be promoted through such receptor clustering. The integrin most extensively studied in the context of HAdV entry is α_v_β_3_ [22], although other integrins, such as α_v_β_5_, α_v_β_1_ and α_3_β_1_ [22, 56] can also function in the same manner.

The first crystal structure of the integrin α_v_β_3_ ectodomain showed a bent conformation, with a globular “head” where the N-terminals of the two subunits meet, then separating into two more loosely associated “legs” [57]. The α_v_ head consists of a seven-bladed β-propeller fold, while a metal ion-dependent adhesion site (MIDAS) of the β subunit mediates the binding between two legs at the “knee”, contributing to the conformational change between bent and open conformation via shifts in the position of loops. The RGD motif-binding site is located in the head of the β subunit.

Integrins bind to the RGD motif on the flexible HAdV penton base loop (with the exception of HAdV-40 and 41, which lack the RGD motif and interact with integrins via an unknown mechanism [24]). While the pentameric penton base consists of five pentons and hence contains five RGD-containing loops, it is highly improbable that five integrins could bind the penton simultaneously due to steric clashes [58]. However, given the importance of integrin clustering for HAdV entry signalling, binding of multiple integrins is expected, probably with variable stoichiometries in solution. Given this asymmetry and heterogeneity, it is unlikely that high-quality crystals of a single type of HadV-integrin complex can be obtained, and electron microscopy is, therefore, the method of choice, as one can distinguish different complexes and process them separately. To date, two studies of whole virus complexes with different integrin receptors [59, 60], and one of monomeric penton base in complex with the integrin [58] have been published.

The imposition of icosahedral symmetry and lack of high-resolution structures of the integrin and penton base to dock into the density at that time meant that it was initially reasoned that a full occupancy of five integrins would be present around the penton. This had been based on structures of HAdV-12 (group A) and HAdV-2 (group C) in complex with integrin α_v_β_5_ at a resolution of about 20 Å, based on a ring of density above the pentamer and SPR measurements showing binding of 4.2 integrins per HAdV-2 pentamer [59]. However, after high-resolution integrin and penton base structures had been published it was recognised that at most four integrins, seen in an extended conformation and in different orientations, could fit around the HAdV-12 penton without steric clashes. The more flexible and extendable RGD loop of HAdV-2 penton was speculated to potentially accommodate five integrins [60].

It was also suggested that the fourth integrin would require a conformational shift in the penton base against the inter-domain “twist”, and that this change may be responsible for the penton’s disassociation from the capsid during HAdV entry and release of the fibre knob [60]. The different integrin orientations and varying occupancies contribute to asymmetry, making the complex structure more challenging to solve with the imposition of symmetry.

Single-particle reconstruction of negative-stain EM monomeric HAdV-9 (group D) penton base insertion domain only integrin α_v_β_3_ showed that a variety of integrin conformations could bind the penton, from bent to extended [58]. Moreover, the monomeric penton was shown to bind the integrin in a number of different locations at the integrin head, showing the interactions to be more varied than expected (see Fig. 3b). It is unclear as yet if this variability is present in the more sterically constrained conditions of virus binding to host cell, and if the integrin conformational flexibility is restrained by the presence of transmembrane and intracellular domains.

## Outlook

HAdVs are remarkable in the diversity of attachment receptors they employ, which is a contributing factor to their broad cellular tropism. Studies of their receptor-virus interactions using structural biology techniques have significantly advanced our understanding of HAdV attachment and entry. This knowledge can be used both in drug design, to combat the virus as a pathogen, and in rational gene vector development, to enhance the virus as a therapeutic agent. Prospects for the field include addressing the imbalance in data on particular receptors: while some of the attachment receptors have been extensively studied in their viral context (e.g. CAR, CD46), there is much less information available on others. Moreover, the integrin entry receptors, representing the second step of HAdV infection, remain to be examined more closely, as we lack a detailed view of the contacts that are being formed in this complex, and we also lack an understanding of how the geometry of binding dictates the stoichiometry of the interaction.
